# Evaluating the course of the saphenous vein and nerve for risk assessment in the suture button technique

**DOI:** 10.1038/s41598-020-80556-y

**Published:** 2021-01-08

**Authors:** Tomo Hamada, Hidenori Matsubara, Toshifumi Hikichi, Hiroyuki Tsuchiya

**Affiliations:** Department of Orthopaedic Surgery, Graduate School of Medical Sciences, Kanazawa University, 13-1 Takara-machi, Kanazawa, 920-8641 Japan

**Keywords:** Anatomy, Risk factors

## Abstract

The suture button technique can cause damage to the saphenous vein and nerve. We examined the location and course of the great saphenous vein using magnetic resonance imaging and determined its position at 10, 20, 30, or 40 mm proximal to the tibial plafond. We divided the region from the anterior to the posteromedial tibial edges into segments A, B, C, D, and E, and compared baseline data and vein parameters between 56 healthy (group H) and 296 symptomatic limbs (group D). At 10, 20, 30, and 40 mm proximal to the tibial plafond, segments A (53.4%), B (45.7%), C (50.0%), and D (52.6%), respectively, had the highest probability of the presence of the great saphenous vein. The mean angle of the great saphenous vein from the distal anterior to the proximal posterior side of the tibia in relation to the tibial axis was 32.4° ± 4.8°. There were no significant differences between groups H and D. These findings indicate that the position of the saphenous vein and nerve should be determined prior to performing the suture button technique on the medial side of the tibia. This can be achieved under direct visualization through a small skin incision or via ultrasound.

## Introduction

The ankle is one of the most frequently fractured sites. Between 13 and 23% of patients experience tibiofibular ligament injury as a complication of ankle fracture^1,2^. Reduction of a precise articular surface and syndesmosis has been indicated as an important predictive factor for overall functional outcome^3–5^. Thus, reconstruction of unstable tibiofibular ligaments is important for a satisfactory therapeutic outcome. The suture button technique is one of the methods for reconstructing tibiofibular syndesmosis, in which the tibia and fibula are fixed using strong sutures and buttons. First, a burr hole is drilled from the lateral side of the fibula to the medial side of the tibia. Then a long needle with a pull-through suture is passed through this hole and used to affix the medial button to the medial tibia. Finally, the fibula and tibia are brought together and affixed with medial and lateral buttons. Many studies have reported that the suture button technique results in better outcomes than the conventional method of tibiofibular fixation using a syndesmosis screw^6–9^. However, because the suture button technique entails affixing the medial button to the medial tibia after passing it through the bone, there is a risk of damage to the great saphenous vein and saphenous nerve. Anatomical studies have indicated that that risk is high, which indicates that it is important to identify the location and course of both the great saphenous vein and saphenous nerve^10,11^. To the best of our knowledge, no large-scale studies about the course of the great saphenous vein and saphenous nerve have been conducted. It is widely known that the saphenous nerve runs along the great saphenous vein. Here, we describe our investigation of the course of the great saphenous vein and saphenous nerve in the limbs of living persons through examination of the location and course of the great saphenous vein using ankle magnetic resonance imaging (MRI).

## Methods

The study protocol was approved by the Medical Ethics Committee of the Kanazawa University Advanced Science Research Center (Approval Number: AP-184005). The requirement of informed consent was waived by the Medical Ethics Committee of the Kanazawa University Advanced Science Research Center. The study was conducted according to the relevant guidelines and regulations.

### Study design and population

This retrospective study included 288 individuals (352 limbs) who underwent foot or ankle MRI in our hospital between April 2005 and January 2019. The following individuals were excluded: patients with a pathophysiology that may indicate changes in the course of their blood vessels, those younger than 18 years of age, patients with osteoarthritis, those with deformations of the lower extremities, patients with a history of para-articular surgery of the ankle, those with space-occupying lesions in the medial or dorsal foot, and patients with swelling of the soft tissue, such as those with a history of para-articular fracture of the ankle. This study also included asymptomatic (healthy) limbs (56 limbs) that were imaged for the purpose of comparison.

### Imaging studies and data analysis

**Figure 1:**
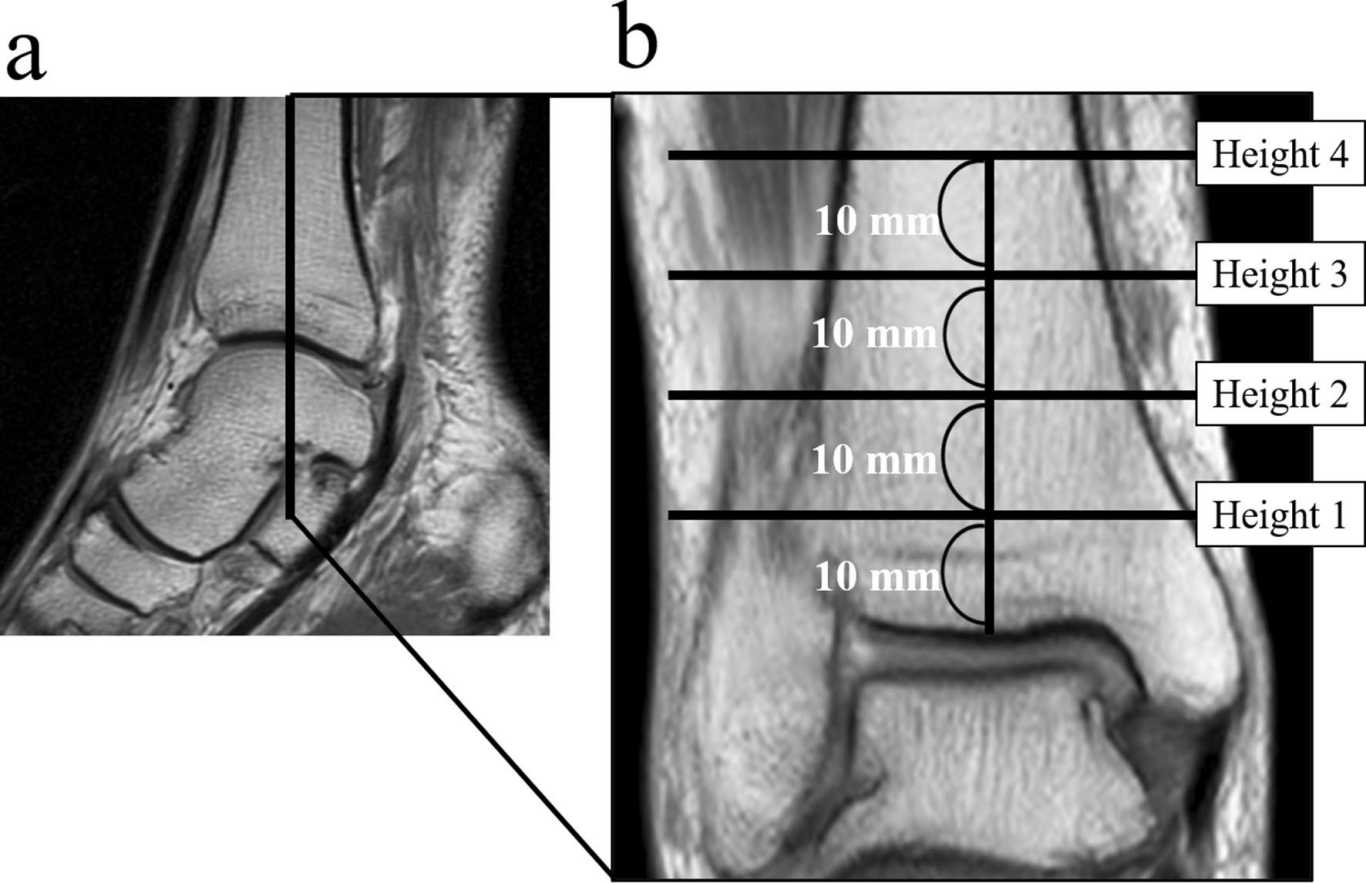
Axial images perpendicular to the coronal images used to determine the location of the great saphenous vein. (**a**) The coronal image is set using sagittal images. (**b**) The tibial axis is set using coronal images, and heights 1–4 were located 10, 20, 30, or 40 mm proximal to the tibial plafond.

All images were obtained using the 1.5 T or 3.0 T MRI scanner (Signa; GE Medical Systems, Milwaukee, WI) in our hospital. All individuals were imaged in the supine position with their ankle joint in the neutral position. The slice angles and imaging parameters (e.g. T1 or T2) varied according to the objective of the imaging studies and the specific sites being imaged, but all image widths were 4-mm sections. In each individual, measurements were made using the image (T1, T2*, and T2STIR) in which the great saphenous vein was most clearly visible. The actual images used were as follows: T1 = 104 limbs, T2* = 192 limbs, and T2STIR = 56 limbs. MRI images were produced using 3D analysis software (Aquarius Net Viewer, TeraRecon, San Mateo, CA). The tibial axis was set using coronal images, and it was determined to be at a height of 10, 20, 30, or 40 mm proximal to the tibial plafond (10 mm: height one, 20 mm: height two, 30 mm: height three, and 40 mm: height four; Fig. 1a,b). Previous reports on the suture button technique determined these heights in consideration of past experiences with the technique as well as manufacturers’ recommendations^6,8,10,11^.

**Figure 2:**
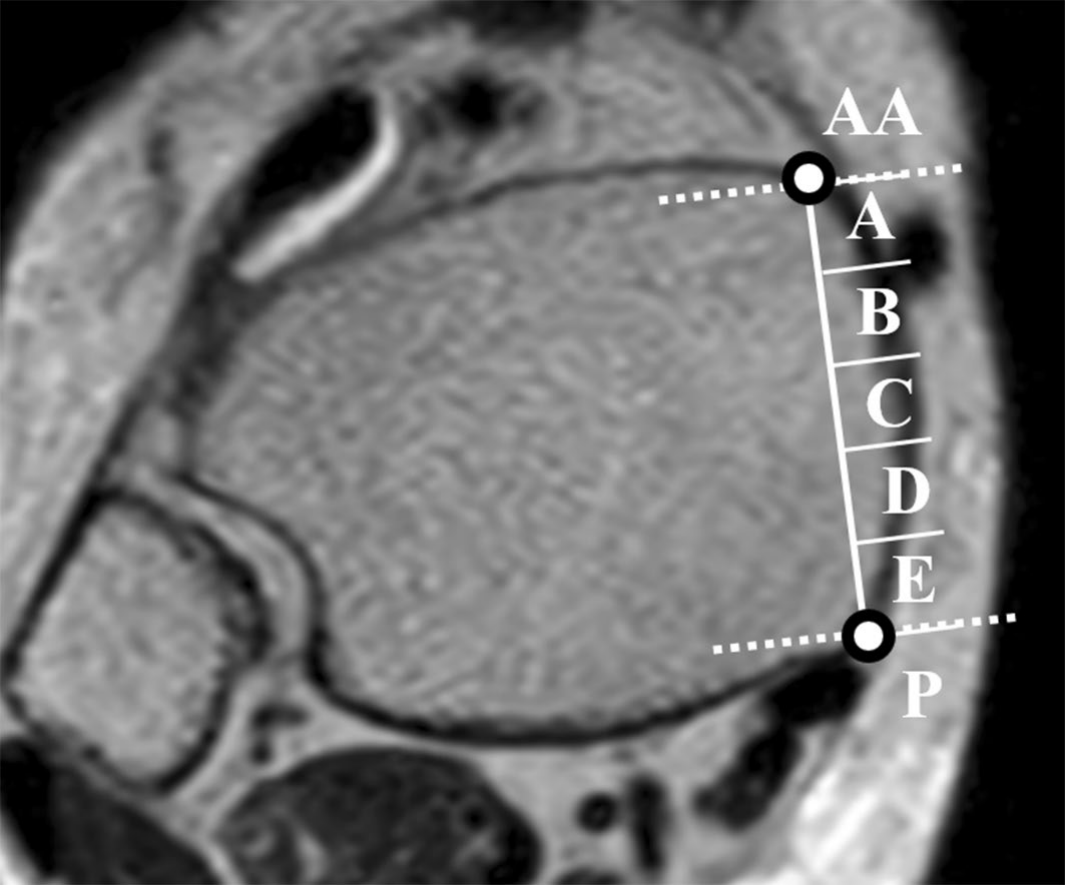
Seven segments used to determine the location of the great saphenous vein. Using an axial image, we divided the region from the anterior edge of the tibia to the posteromedial edge into five segments (A, B, C, D, and E), and designated the segment that was anterior to the anterior edge as ‘AA’ and the segment that was posterior to the posterior edge as ‘P.’ The course of the great saphenous vein through these segments at heights 1–4 described in Fig. 1 was evaluated.

**Figure 3:**
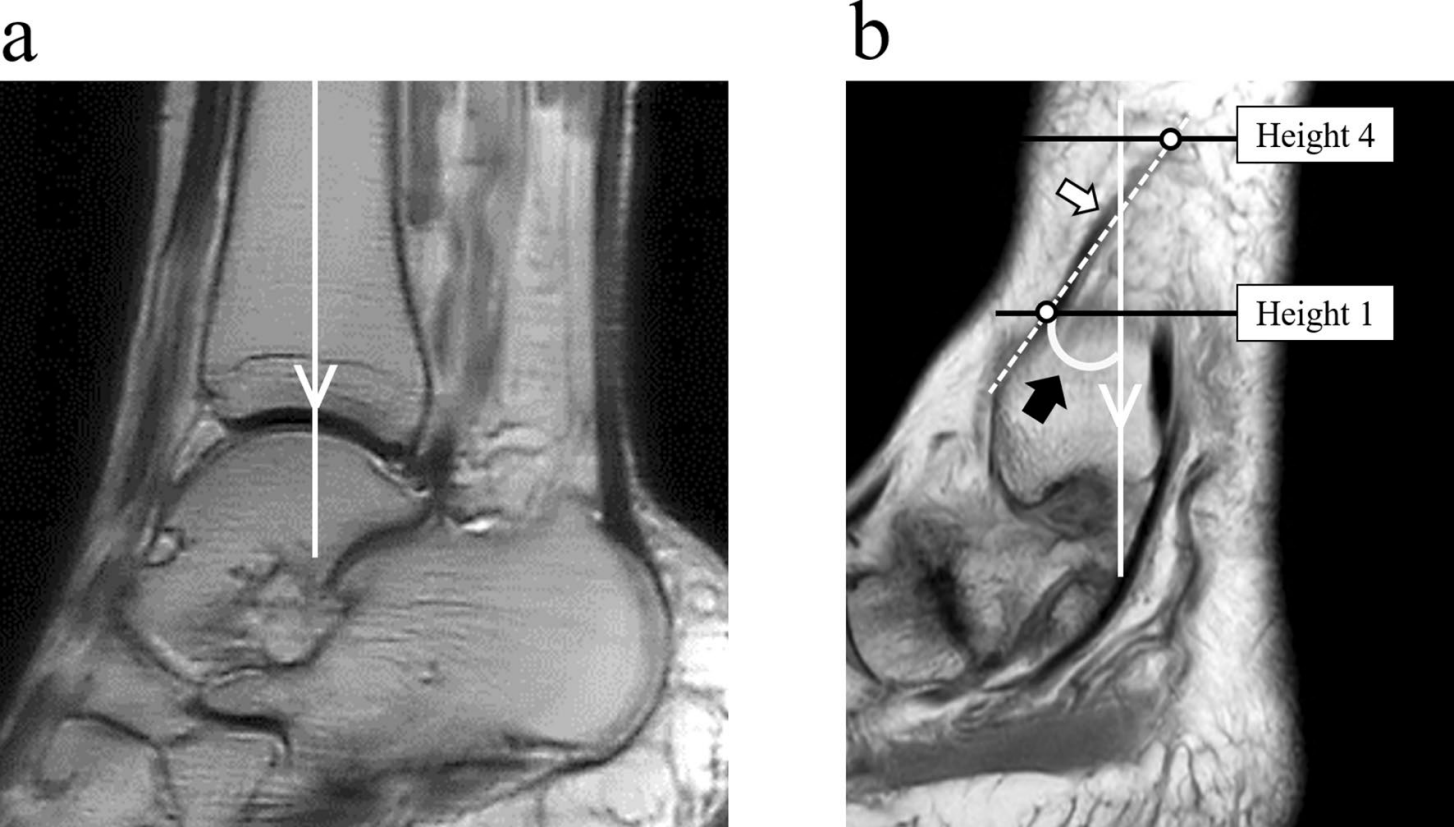
Sagittal images of the ankle joint based on the tibia. (**a**) The tibial axis is indicated. (**b**) The course of the great saphenous vein is indicated from height one to height four, and the angle formed between this line and the tibial axis is observed. White arrow: Great saphenous vein. Black arrow: Angle of the course of the great saphenous vein.

We examined the location of the great saphenous vein using axial images that were perpendicular to the coronal images. We defined the inflection point between the tibial anterior surface and tibial medial surface as the anterior edge and that between the tibial medial surface and tibial posterior surface as the posterior edge. We also divided the region from the anterior edge of the tibia to the posteromedial edge into five segments (from the anterior edge: A, B, C, D, and E), and designated the segment that was anterior to the anterior edge as ‘AA’ and the segment that was posterior to the posterior edge as ‘P’ (Fig. 2). We investigated the course of the great saphenous veins as follows. By using sagittal images of the tibial axis, we established a line that indicated the course of the great saphenous vein from height one to height four, and observed the angle formed between this line and the tibial axis (angle of course; Fig. 3a,b).

We also compared the baseline data (e.g. age, sex, and right versus left side) and the location and course of the great saphenous vein between 56 limbs (in which the vein was incidentally imaged, group H) and 296 limbs (in which imaging was done for some symptoms, group D).

### Statistical analysis

All results are indicated as a mean ± standard deviation. Significant differences were determined using the Pearson chi-square test and non-parametric Mann–Whitney U test. All statistical analyses were performed using the Social Science Statistics Package version 23.0 (IBM Corp., Armonk, NY). Statistical significance was set at P < 0.05.

## Results

The participants comprised 136 men and 216 women, and the mean age was 51.9 (range 18–87) years. The reasons for performing the imaging studies were as follows: ankle pain = 113 limbs (MRI indicated no obvious abnormal findings), para-articular damage to the tendons and ligaments of the ankle = 56 limbs, hindfoot symptoms such as heel pain = 45 limbs, midfoot/forefoot symptoms = 31 limbs, local intraosseous swelling (no influence on the surrounding soft tissue) = 23 limbs, space-occupying lesion on the lateral ankle = 20 limbs, osteochondral dissecans of the ankle = seven limbs, intraarticular tumor of the ankle = one limb, and asymptomatic (healthy) limbs = 56 limbs.

**Figure 4:**
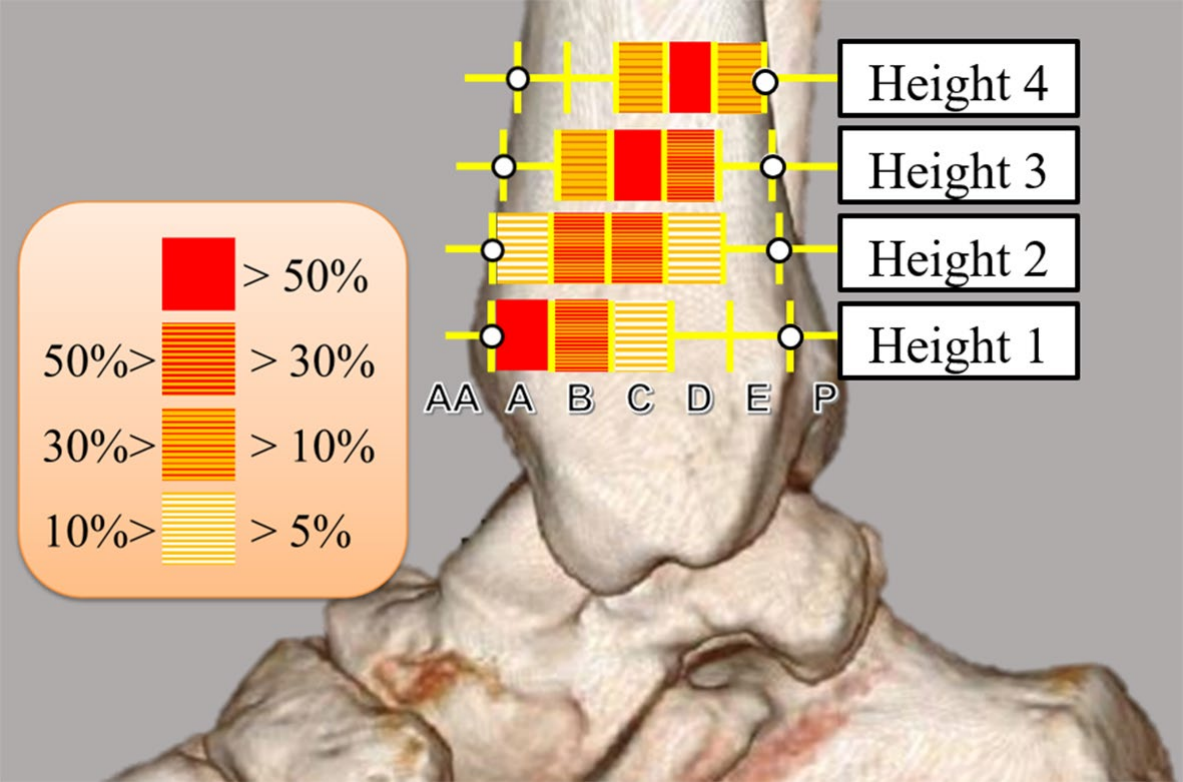
Prevalence of the great saphenous vein at each location. The prevalence of the great saphenous vein in each segment (AA-P) at each height (height 1–4) is shown.

The position of the great saphenous veins in each tibial segment is shown in Table 1. Figure 4 shows each segment in a separate color to indicate the degree of risk. The mean angle at which the great saphenous vein followed a course from the distal anterior side to the proximal posterior side in relation to the tibial axis was 32.4° ± 4.8° (15.2°-47.8°). No significant differences were found between group H and group D in terms of any of the variables we investigated (Table 2).

**Table 1:** Location of the great saphenous vein.

Tibial segment^a^	Tibial axis height			
	Height 1 (10 mm)	Height 2 (20 mm)	Height 3 (30 mm)	Height 4 (40 mm)
AA	14 (4.0%)	3 (0.9%)	0	0
A	188 (53.4%)	32 (9.1%)	5 (1.4%)	2 (0.6%)
B	117 (33.2%)	161 (45.7%)	48 (13.6%)	7 (2.0%)
C	31 (8.8%)	130 (36.9%)	176 (50.0%)	63 (17.9%)
D	2 (0.6%)	25 (7.1%)	106 (30.1%)	185 (52.6%)
E	0	1 (0.3%)	17 (4.8%)	90 (25.6%)
P	0	0	0	5 (1.4%)

^a^The anterior edge of the tibia to the posteromedial edge was divided into five segments from the anterior edge: A, B, C, D, and E. AA = the segment anterior to the anterior edge and P = the segment posterior to the posterior edge.

**Table 2:** Patient demographics.

	Group H (n = 56)	Group D (n = 296)	*p*-value
Age, years (mean ± SD)	57.6 ± 14.3	50.9 ± 17.3	0.199
Male sex (%)	21 (38)	115 (39)	0.882
Right/left	30/26	164/132	0.770
Position of the great saphenous vein (%)10 mm AA	1 (2)	13 (4)	0.153
A	38 (68)	150 (51)	
B	15 (27)	102 (34)	
C	2 (4)	29 (10)	
D	0	2 (1)	
E	0	0	
P	0	0	
20 mm AA	0	3 (1)	0.263
A	7 (13)	29 (10)	
B	32 (23)	125 (42)	
C	15 (27)	115 (39)	
D	2 (4)	23 (8)	
E	0	1 (0.3)	
P	0	0	
30 mm AA	0	0	0.247
A	0	5 (2)	
B	11 (20)	37 (13)	
C	31 (55)	145 (49)	
D	13 (23)	93 (31)	
E	1 (2)	16 (5)	
P	0	0	
40 mm AA	0	0	0.141
A	0	2 (1)	
B	1 (2)	7 (2)	
C	16 (29)	46 (16)	
D	30 (54)	155 (52)	
E	9 (16)	81 (27)	
P	0	5 (2)	
Tilt of the great saphenous vein, ° (mean ± SD)	32.4 ± 5.0	32.2 ± 3.3	0.384

*SD* standard deviation.

## Discussion

In this study, we determined the trends displayed in the course of the great saphenous vein at heights where the suture buttons are inserted for the purpose of reconstructing ankle syndesmosis. Further, we found that the great saphenous vein is located at a different site in each individual, despite having the same height, and that even slight differences in height result in major differences in the location of the vein.

**Figure 5:**
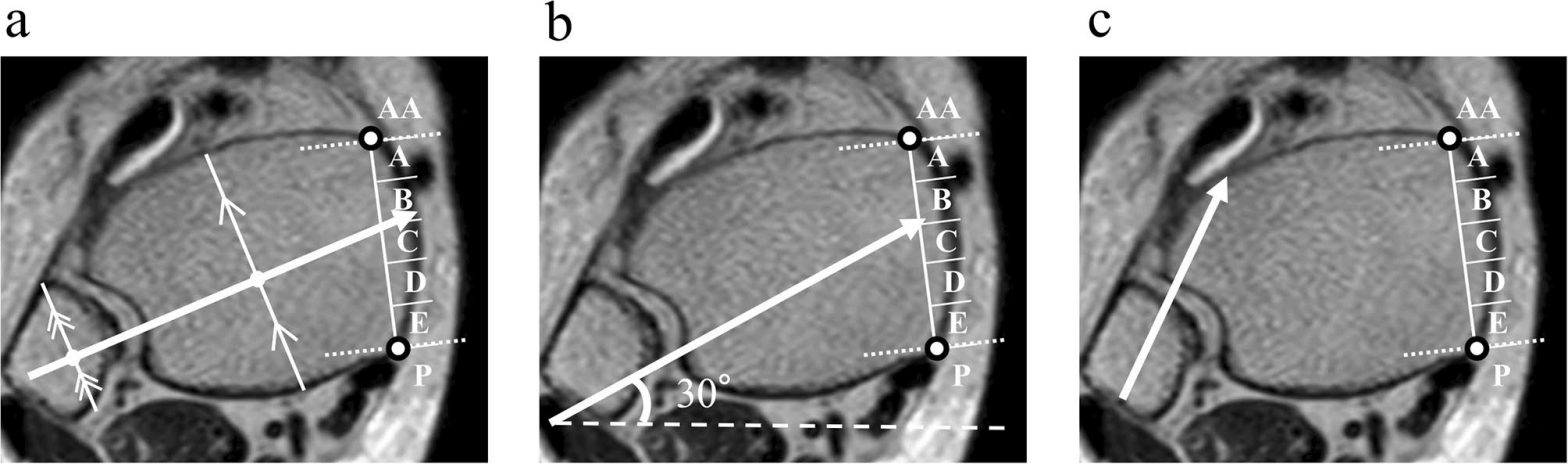
The risk of injuring the great saphenous vein with the center-center technique of suture button insertion. (**a**) The commonly used center-center technique of suture button insertion affects segment B at a height of 10 mm. (**b**) The insertion of suture buttons at an angle of approximately 30° in relation to the frontal plane affects segment B or C at a height of 20 mm. (**c**) When fixation is performed at the anterior tibiofibular ligament attachment site, there is no risk of injury to the great saphenous vein. White arrows: the course of the suture button insertion.

Several studies have pointed out that, from an anatomical perspective, there is a risk of causing damage to the great saphenous vein and saphenous nerve when using the suture button technique. Reb et al.^11^ indicated that at heights of 10, 20, and 30 mm proximal to the ankle joint, lateral images of the suture buttons showed that in 11 of 30 cases (36.7%), the medial tibial cortex apertures were directly in line with the great saphenous vein when the center-center technique was used to drill from the center of the fibula through the center of the tibia. In addition, in over half of these cases (6/11, 55%), the suture button was in contact with the saphenous tissue at a height of 10 mm. Observation of axial images indicated that the center-center technique they utilized corresponded to segment B or C at height one in this study (Fig. 5a). In the present study, there were 117 limbs (33.2%) in segment B at height 1, and there was an extremely high probability of great saphenous vein crossing at this height. Pirozzi et al.^10^ reported that based on the insertion of suture buttons at an angle of approximately 30° in relation to the frontal plane and at a height of 20 mm proximal to the ankles of 20 limbs of fresh cadavers, 11 suture buttons (55.0%) were inserted with entrapment of a neurovascular structure. This method corresponds chiefly to segment B or C at the height two in our study (Fig. 5b). With 161 limbs (45.7%) in segment B and 130 limbs (36.9%) in segment C, these two segments were the most frequently utilized segments in which the saphenous vein course was present. The results of our study support the findings of previous studies. Our study is, to the best of our knowledge, the first large-scale imaging study reporting the high risk of injuring the great saphenous vein and saphenous nerve when using the common suture button insertion method.

Our study’s findings indicated that a safer method of insertion should aim for the posterior side of the medial tibia at a height of 10 mm proximal to the tibial plafond, as measured from the tibial plafond. In addition, aiming for the anterior side of the medial tibia at 20 mm or 30 mm proximal to the tibial plafond would avoid the sites where the courses of the great saphenous vein and saphenous nerve are most likely to be located. However, there remains doubt as to whether this insertion angle will result in stabilization of the tibiofibular syndesmosis. Some studies have also reported that subtle differences in the angle of tenaculum clamp placement have demonstrated substantial fibular malreduction^12,13^. Even with these suggested insertion locations, there remains a risk of approximately 10% of encountering the course of the great saphenous vein. In their comparison of fixation methods that utilize suture buttons in cases of anterior inferior tibiofibular ligament (AITFL) mono-injury, Teramoto et al.^14^ reported that when using a method of anatomical reconstruction in which fixation is performed from the posterior cortex of the fibula to the anterolateral edge of the tibia at the AITFL attachment site (Fig. 5c), they were able to perform similar dynamic stabilization of the intact specimens. This fixation method was found to eliminate the risk of injury to the great saphenous vein and saphenous nerve, but the study parameters were limited to AITFL mono-injury. Therefore, to prevent injury to the saphenous nerve and great saphenous vein, visualization of the medial suture button insertion site is recommended. This can be achieved under direct visualization through a small skin incision or via ultrasound.

When using the suture button technique, it is necessary to affix the medial button to the medial side of the tibia. The medial button is oblong or rectangular, and if it is affixed perpendicular to the courses of the great saphenous vein and saphenous nerve, there is the risk of interposition of these structures between the medial button and bone. Additionally, during load or motion of the ankle joint, the risk of impingement of between the medial button and great saphenous vein or saphenous nerve may increase. Thus, we believe that the final fixation angle of the medial suture button is important to consider. However, as far as we were able to determine, there are no recommendations regarding the angle of the medial button. We found that the great saphenous vein follows a course that is a mean of 32.4° anterior to the tibial axis. We believe that it is preferable for the angle of placement of the medial button to be parallel to the angle of the course of the great saphenous vein.

This study has several limitations. First, the measurements were made using MRI images that were obtained for some symptom in many cases. We excluded those cases that we believed may have some effect on the course of the great saphenous vein, and our comparison of affected limbs to healthy limbs did not show significant differences between the two groups. However, we cannot rule out the possibility that various diseases have an effect on the course of the great saphenous vein. Second, the original imaging parameters and angles were different in each individual. In order to minimize the effect of this factor, we were able to integrate the measurement angles using three-dimensional image processing software, but the imaging parameters differed in each case. Third, we investigated the location and course of the great saphenous vein only, and did not include data regarding the course of the saphenous nerve. However, injury to the saphenous nerve is an important complication of the suture button technique. Marsland et al.^15^ reported that the saphenous nerve and saphenous vein were within 1 mm of each other in 71% of the ankles studied. The results of our study indicate that the course of the great saphenous vein differs between individuals, suggesting that the course of the saphenous nerve may also differ. Last, this study included the limbs of Asian individuals only. Studies of other ethnicities may reveal differing results because of differences in physique.

We verified that the location of the great saphenous vein around the ankle joint displays great variation among individuals, indicating a high risk of injury to the great saphenous vein and the saphenous nerve when using this prominent method to insert suture buttons. Therefore, visualization of the insertion location should be performed prior to insertion of the medial suture button, which can be achieved under direct visualization through a small skin incision or via ultrasound. These results will help surgeons avoid injury to the great saphenous vein and saphenous nerve when performing various procedures on the medial side of the tibia, including the suture button technique.

## Data Availability

All data generated or analyzed during this study are included in this published article.
